# How Checkpoint Inhibitors Are Changing the Treatment Paradigm in Solid Tumors: What Advanced Practitioners in Oncology Need to Know

**Published:** 2016-07-01

**Authors:** Marianne Davies

**Affiliations:** Yale University School of Nursing, Yale Cancer Center, New Haven, Connecticut

## Abstract

The immune system plays an active role in controlling and eradicating cancer. T cells, an essential component of the adaptive immune system, have a number of surface receptors (called "checkpoints") that can help either to sustain activation or suppress T-cell function. Many malignancies have developed ways to exploit these receptors to suppress T-cell function, enabling them to continue to grow. Anticancer immunotherapy in general, and checkpoint inhibitor therapy specifically, is a unique approach to cancer treatment that strives to harness the body’s own immune system to generate an adequate response against cancer cells. Several checkpoint inhibitors are approved for the treatment of metastatic melanoma, non–small cell lung cancer, and renal cell carcinoma. These and other agents in this class are being investigated for their safety and efficacy in a variety of solid and hematologic malignancies. Advanced practitioners (APs) play a critical role in caring for patients treated with checkpoint inhibitors. It is essential for APs to be aware of the mechanism of action of these agents, patterns of response seen with this type of therapy, and presentation of immune-related adverse events related to these agents to ensure timely and successful treatment. Rapid evaluation/diagnostics and treatment are essential for optimal management and prevention of end-organ disease, and treatment of immune-related adverse effects requires a multidisciplinary approach.

## How Checkpoint Inhibitors Are Changing the Treatment Paradigm in Solid Tumors: What Advanced Practitioners in Oncology Need to Know

The immune system, comprising the innate and adaptive systems, plays an active role in controlling and eradicating cancer. The innate immune system (including macrophages, neutrophils, and immature dendritic cells) recognize aberrant cells and are phagocytic. This response is usually rapid and associated with inflammation, but this system does not have specificity and does not generate immunologic memory.

The adaptive immune system (including T lymphocytes, B lymphocytes, and antigen-presenting cells), primarily through T cells, is predominantly involved in eradicating the body of cancer. B cells recognize antigens from tumors and develop antibodies that bind to circulating antigens. T cells differentiate into a number of different subtypes, which have specificity for certain antigens, either "self-"antigens or "foreign" antigens. CD4 T cells make cytokines to help amplify the immune system, and CD8 T cells process foreign antigens and stimulate cell destruction.

The immune response generated against aberrant cells such as tumor cells is generally a more specific and slower response. The adaptive immune system develops immunologic memory. Several ligands and receptors that either enhance or suppress T-cell activity have been identified, and they are classified as checkpoint pathways (Table 1).

**Table 1:**
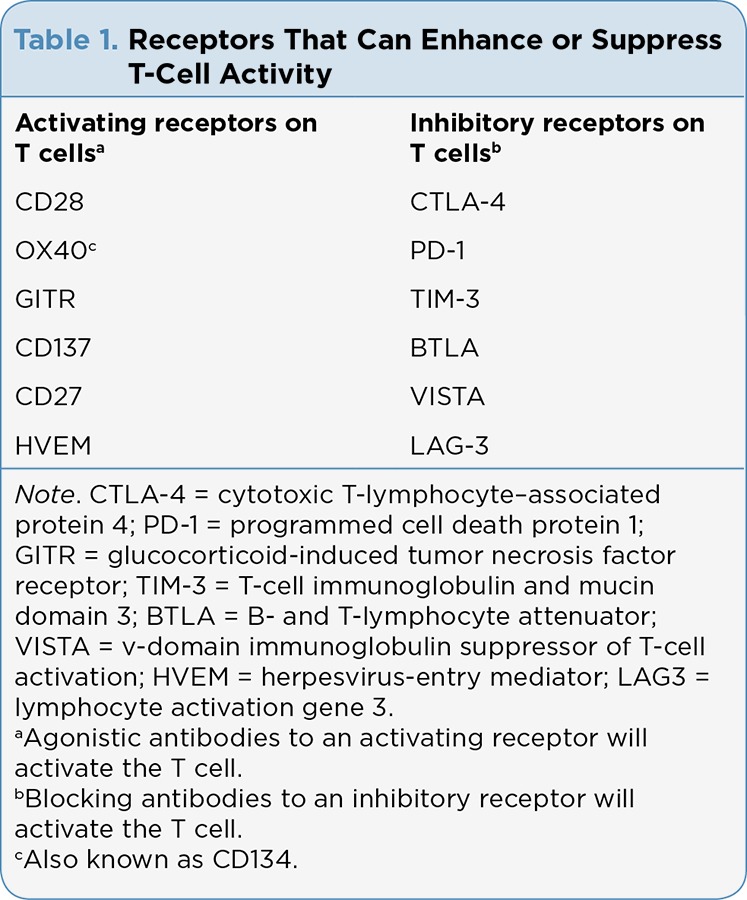
Receptors That Can Enhance or Suppress T-Cell Activity

Initially when a tumor develops, there is an elimination phase in which the tumor is recognized as foreign and destroyed by the immune system. If the tumor persists, a state of equilibrium develops in which the immune system can destroy only part of the tumor, but the tumor does not grow. Eventually, however, the tumor "escapes" by activating the checkpoint pathway and developing other properties that allow it to evade the immune system and continue to grow (Mittal, Gubin, Schreiber, & Smyth, 2014; Schreiber, Old, & Smyth, 2011).

**Figure 1:**
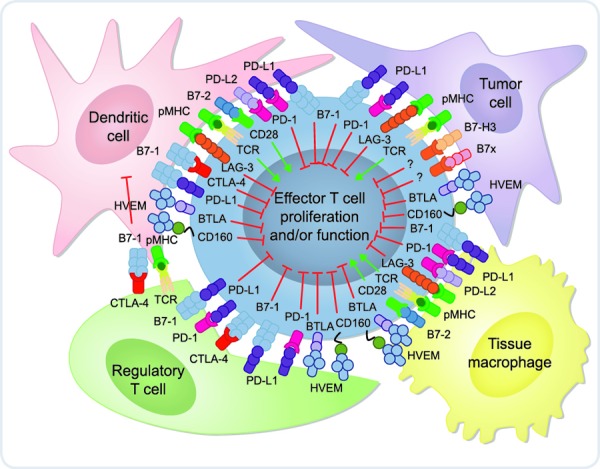
T-cell inhibitors of the immunoglobulin superfamily. Reprinted with permission from Pentcheva-Hoang et al. (2009).

Checkpoint proteins such as cytotoxic T-lymphocyte–associated antigen-4 (CTLA-4) and programmed cell death protein 1 (PD-1) are inhibitory receptors expressed on the T-cell surface after T-cell activation (Figure 1; Pentcheva-Hoang, Corse, & Allison, 2009). Both of these receptors have been identified as key checkpoints in tumor evasion. CTLA-4 works during the initial phases of T-cell activation. Its primary function is to downregulate T-cell activation in lymphatic tissues early in the immune response. PD-1 is expressed on T cells, B cells, and natural killer cells; it limits T-cell activity in the peripheral tissues during cell-mediated immune responses.

The PD-1 receptor interacts with two ligands, PD-L1 and PD-L2, which are expressed on tumor cells and other cells. PD-L1 is often upregulated in solid tumors. The interaction between the ligand and the receptor is necessary to maintain normal homeostasis in the setting of infection or inflammation to prevent autoimmunity or overproliferation of the T cell. In the setting of tumor, however, the receptor-ligand interaction provides immune escape by suppressing T-cell function and enabling the tumor to continue to proliferate (Disis, 2014; Drake, Lipson, & Brahmer, 2014; Drake, Jaffee, & Pardoll, 2006; Nirschl & Drake, 2013; Ohaegbulam, Assal, Lazar-Molnar, Yao, & Zang, 2015; Pardoll, 2012; Taube et al., 2014).

## MECHANISM OF ACTION AND IMPACT ON IMMUNE SYSTEM

Anticancer immunotherapy is a unique approach to cancer treatment that strives to harness the body’s own immune system to generate an adequate response against tumors (Mellman, Coukos, & Dranoff, 2011). T-cell activation is required for an effective antitumor immune response. Once CTLA-4 and PD-1 are activated, they downregulate T cells. Tumor cells can exploit these pathways to promote and maintain suppression of T-cell function, allowing the tumor to grow.

The immune checkpoints CTLA-4 and PD-1 are targets for new and emerging drug development, called checkpoint inhibitors (Postow, Callahan, & Wolchok, 2011, 2015). Checkpoint inhibitors have led to advances in the treatment of several solid tumors, including metastatic melanoma (MM), non–small cell lung cancer (NSCLC), and renal cell carcinoma (RCC). (Note that checkpoint inhibitors also are being investigated for treatment of a variety of other solid and hematologic malignancies, but for the purposes of this article, the discussion will be confined to their use in the treatment of MM, NSCLC, and RCC.)

**Figure 2:**
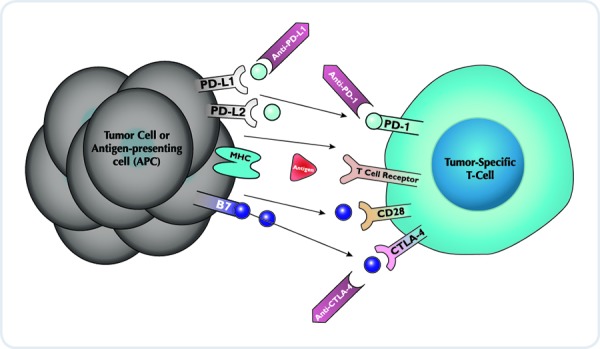
Immune checkpoint blockade. Adapted from Drake et al. (2014).

Checkpoint inhibitors, such as anti–CTLA-4 monoclonal antibodies (mAbs), anti–PD-1 mAbs, and anti–PD-L1 mAbs, restore T-cell activity by blocking the receptor-ligand bond responsible for creating a state of immune tolerance (Figure 2; Blank, 2014; Langer, 2015; Leach, Krummel, & Allison, 1996; Pardoll, 2012; Postow et al., 2015; Sharma, Wagner, Wolchok, & Allison, 2011; Tarhini, Lo, & Minor, 2010). In effect, the checkpoint inhibitors release the brakes on T cells, allowing for sustained T-cell activation, so these immune cells can recognize and attack tumor cells (Hodi et al., 2010).

## CHECKPOINT INHIBITORS: INDICATIONS AND SAFETY

There are a number of checkpoint inhibitors approved and/or in late stages of development for the treatment of MM, NSCLC, and RCC. The evaluation of these agents is an extremely active area of research. The following section will highlight some of the data published/presented on these agents. It is important to note, however, that this section is not meant to be an all-inclusive review.

**Anti–CTLA-4 Agents**

Ipilimumab (Yervoy) was approved in 2011 for the treatment of unresectable or metastatic melanoma (Table 2). The approved dose is 3 mg/kg intravenously (IV) over 90 minutes and is administered every 3 weeks, for a maximum of four doses (weeks 1, 4, 7, and 10; Bristol-Myers Squibb, 2015b). Re-induction may be a consideration in patients with disease progression after a period of stable disease for 3 months after induction therapy.

**Table 2:**
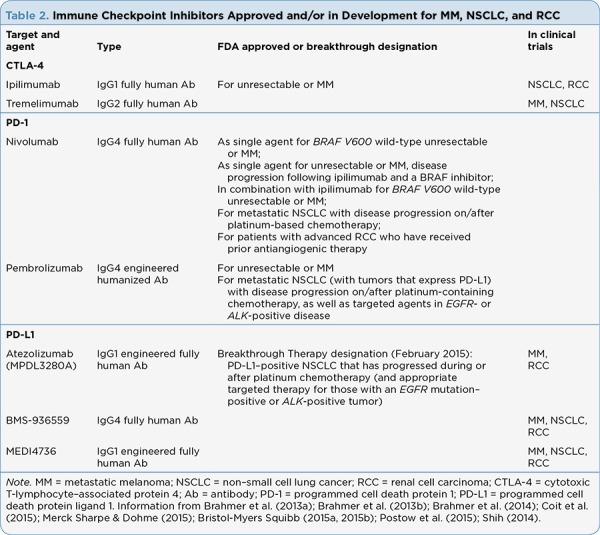
Immune Checkpoint Inhibitors Approved and/or in Development for MM, NSCLC, and RCC

Ipilimumab was approved based on two phase III studies evaluating patients with advanced melanoma. One study evaluated the effect of ipilimumab in previously untreated patients, and the other evaluated its effect in a treatment-experienced population (Robert et al., 2011; Hodi et al., 2010). Significant survival benefits and prolonged stable disease were seen with ipilimumab in both studies, with approximately 46% (for treatment-experienced patients) and 47% (for treatment-naive patients) survival rate at 1 year, and approximately 24% (treatment-experienced) and 28% (treatment-naive) alive at 2 years (Hodi et al., 2010; Robert et al., 2011). Long-term follow-up has demonstrated 20% of patients with at least 3-year survival, with the longest survival thus far over 10 years (Prieto et al., 2012; Schandendorf et al., 2015; Wolchok et al., 2013a).

Patients with NSCLC and small cell lung cancer have also had improved progression-free survival (PFS) with ipilimumab compared with carboplatin and paclitaxel (Lynch et al., 2012; Reck et al., 2013); however, it is still in clinical trials for these diseases.

Tremelimumab is currently being investigated for the treatment of several solid tumors, including melanoma and lung cancer (Tarhini, 2013; Ribas et al., 2013).

**Anti–PD-1 Agents**

Anti–PD-1 checkpoint inhibitors provide high response rates, shorter times to response, and durable responses with a more favorable toxicity profile compared with anti–CTLA-4 agents.

*Nivolumab:* Nivolumab is currently approved for the treatment of MM, metastatic NSCLC, and RCC. The approved dose is 3 mg/kg IV over 60 minutes, given every 2 weeks, except when used in combination with ipilimumab; in that instance, the dosing is 1 mg/kg, followed by ipilimumab on the same day, every 3 weeks for four doses, then 3 mg/kg every 3 weeks (Bristol-Myers Squibb, 2015a). Survival rates in patients with melanoma were approximately 62% at 1 year and 43% at 2 years (Sznol et al., 2013; Topalian et al., 2014).

In a phase III, randomized study of 272 patients with squamous NSCLC, previously treated with a platinum-based regimen, patients treated with nivolumab demonstrated a significant survival benefit over docetaxel at 1 year (42% vs. 24%, respectively) regardless of PD-L1 status (Brahmer et al., 2015). A study evaluating the efficacy of nivolumab in patients with nonsquamous NSCLC showed a 50% survival at 1 year compared with 39% in patients treated with docetaxel (Paz-Ares et al., 2015). Expression of PD-L1 has been associated with improved overall survival (OS; Gettinger et al., 2014; Paz-Ares et al., 2015).

Patients with metastatic RCC also have demonstrated improvement in overall response rate (ORR) of 20% to 29% with nivolumab (Drake et al., 2013; Motzer et al., 2014; Topalian, 2012b). A phase III trial comparing nivolumab with everolimus (Afinitor) in previously treated patients with RCC was recently stopped early because it met its primary endpoint of improved OS.

*Pembrolizumb:* Pembrolizumab is approved for the treatment of patients with metastatic melanoma and is dosed at 2 mg/kg IV over 30 minutes and administered every 3 weeks (Merck Sharpe & Dohme, 2015). In a phase I trial, patients who were ipilimumab-naive had a 40% ORR, and those who were previously treated had an ORR of 28% (Ribas et al., 2014). A recent phase III study compared two dosing schedules of pembrolizumab (every 2 weeks vs. 3 weeks) with ipilimumab (every 3 weeks; Robert et al., 2015). The estimated 6-month PFS was highest in the pembrolizumab every-2-week dosing schedule (47.3%) compared with 46.4% when pembrolizumab was given every 3 weeks and 26.5% for those taking ipilimumab. The study was stopped early because of the survival and response benefits seen with pembrolizumab (Robert et al., 2015).

In November 2015, the FDA approved the use of pembrolizumab in patients with metastatic NSCLC (with tumors that express PD-L1). Data evaluating the use of pembrolizumab in patients with advanced NSCLC showed an ORR of 18% for previously treated patients and 24.8% for treatment-naive patients (Garon et al., 2015). Data have suggested an improved response in patients who have higher PD-L1 expression. Higher response rates have been noted in patients who are current smokers/former smokers vs. never smokers (26% and 8%, respectively; Garon et al., 2013).

**Anti–PD-L1 Agents**

At least three anti–PD-L1 agents are currently in clinical trial development. In a phase I study of atezolizumab (MPDL3280A; Tecentriq) in metastatic melanoma, ORR was 39%, with 43% demonstrating a PFS of 24 weeks (Hamid et al., 2013b). Patients with RCC had an ORR of 13%, and 53% demonstrated PFS at 24 weeks (Cho et al., 2013; Herbst et al., 2013). Patients with NSCLC who had PD-L1 expression had an ORR of 38% in a phase II trial comparing atezolizumab with docetaxel (13% ORR; Spira et al., 2015).

Two additional agents in this class are BMS-936559 and MEDI4736. Tumor regression was seen in multiple tumor types including MM, NSCLC, and RCC treated with MEDI4736 (Lutzky et al., 2014).

It is important to point out that PD-L1 may not be expressed on the entire tumor cell surface, so testing for expression of this ligand may not be consistent. Some studies have shown higher responses in patients whose tumors express moderate to high levels of PD-L1 compared with tumors with low expression (Antonia et al., 2014b; Garon et al., 2013; Herbst et al., 2015; Topalian, Drake, & Pardoll, 2012b; Weber, Kähler, & Hauschild, 2012), whereas responses have also been reported in patients with undetectable levels of PD-L1 (Antonia et al., 2014b; Garon et al., 2013; Weber et al., 2012). Differences in testing assays among these studies may be one reason for these variable outcomes.

Several trials exploring combinations of checkpoint inhibitors are currently underway, as they affect separate and nonoverlapping pathways. There are indications that combining checkpoint inhibitors may achieve a synergistic effect (Selby et al., 2013; Sznol et al., 2014; Wolchok et al., 2013b).

The combination of ipilimumab and nivolumab is currently approved for the treatment of BRAF V600 wild-type unresectable or MM (Bristol-Myers Squibb, 2015a). A phase III randomized study of ipilimumab and nivolumab vs. single-agent ipilimumab or nivolumab was conducted with 945 previously untreated patients with MM. The combination regimen and nivolumab alone demonstrated significantly longer PFS (11.5 and 6.9 months, respectively) than did ipilimumab alone (2.9 months; Larkin et al., 2015). Severe adverse side effects were increased with the combination group (55%) compared with the ipilimumab monotherapy (27.3%) or nivolumab monotherapy (16.3%) groups.

A study of 46 patients with RCC treated with the combination of ipilimumab and nivolumab demonstrated an ORR of 45%, and 65% of patients demonstrated PFS at 24 weeks (Hammers et al., 2014). Trials evaluating the combination of ipilimumab and nivolumab in NSCLC are now underway.

Data from a phase Ib dose-escalation study combining tremelimumab with MEDI4736 in patients with NSCLC demonstrated clinical activity despite PD-L1 status (Antonia et al., 2015).

Additional studies are exploring the efficacy and safety of combining checkpoint inhibitors with chemotherapy, epidermal growth factor receptor (EGFR) tyrosine kinase inhibitors, other mAbs (e.g., sunitinib [Sutent]/pazopanib [Votrient]), and radiation therapy in NSCLC and RCC (Amin et al., 2014; Antonia, Brahmer, Gettinger, Reardon, & Sampson, 2014a; Rizvi et al., 2014).

## PATTERNS OF RESPONSE

Patterns of response from checkpoint inhibitor treatment differ from the conventional antitumor response seen with standard chemotherapy. Wolchock and colleagues (2009) described four distinct patterns of response:

- Growth of existing metastases attributed to therapy-induced inflammatory infiltrates of activated T cells. This may be associated with pain.
- New lesions may appear during therapy, followed by subsequent regression of lesions.
- Stable disease on first post-treatment scans followed by decline in tumor burden.
- Response after initial increase in tumor burden.

Some patients do not show evidence of disease regression for many weeks after the initiation of checkpoint inhibitor treatment, and some reports of response have been seen up to 12 months after treatment started. Apparent progression, coined "pseudoprogression" or "tumor flare," is thought to be due to local inflammation.

The pattern of response seen with checkpoint inhibitors is usually delayed, which has been attributed to the mechanism of action, as mounting a T-cell response takes time. Because responses can occur slowly or mixed, 12 weeks has been established as the time to first evaluation for ipilimumab. Responses from anti–PD-1 agents and anti–PD-L1 agents are usually seen sooner, as they act at the local level (Pennock, Waterfield, & Wolchok, 2012). These patterns of response have been associated with durable responses in patients (Wolchok et al., 2009).

Conventional Response Evaluation Criteria in Solid Tumors (RECIST) or World Health Organization criteria were designed to evaluate the early effects of conventional therapies (e.g., cytotoxic agents and radiation therapy). However, these criteria do not account for the variable patterns of response that may be seen with the checkpoint inhibitors. As an alternative, criteria were developed and proposed: the immune-related response criteria (irRC; Hoos et al., 2010; Wolchok et al., 2009). These alternative criteria remain under investigation.

## IMMUNE-RELATED TOXICITIES

Checkpoint inhibitors are generally well tolerated, with the most common adverse effects (AEs) being fatigue, decreased appetite, and arthralgias. They are similar to the profile of AEs seen with standard chemotherapy. However, enhancement and restoration of the immune system are associated with unique AEs, referred to as immune-related adverse events (irAEs). Immune checkpoint inhibitors activate the immune system and promote sustained T-cell activity, but the effects of this sustained activation cannot be confined to antitumor effects. This amplification of the immune system can cause T cells to attack healthy tissue, a process referred to as autoimmunity (Di Giacomo, Biagioli, & Maio, 2010; Fecher, Agarwala, Hodi, & Weber, 2013; Weber et al., 2012). This process of inflammation can occur in any organ of the body, but it typically occurs in organ systems that contain significant T cells (Table 3).

**Table 3:**
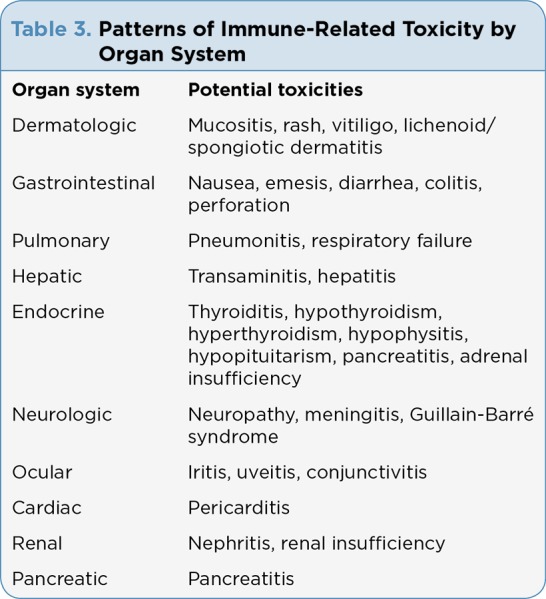
Patterns of Immune-Related Toxicity by Organ System

**Figure 3:**
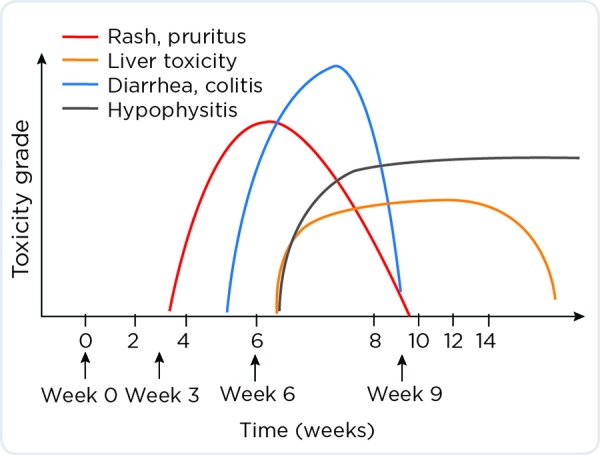
Appearance of immune-related adverse events with checkpoint inhibitors. Reprinted with permission from Weber et al. (2012).

Adverse events may occur during treatment, immediately after treatment, or weeks to months after treatment continuation or discontinuation. The pattern of presentation is variable. The time to onset of irAEs is generally 6 to 8 weeks (Figure 3; Dummer, Maio, & Hamid, 2010; Weber et al., 2012). The delay in onset of AEs is in alignment with the mechanism of action of checkpoint inhibitors, as it takes time to amplify the immune system.

Most irAEs are low grade (grade 1 or 2), with a range of 40% to 60% of patients experiencing irAEs in these grades; grade 3 to 4 toxicity has been noted in up to 6% to 20% of patients across studies (Brahmer et al., 2012; Hamid & Carvajal, 2013a; Hodi et al., 2010; Ibrahim et al., 2011; Lynch et al., 2012; Topalian et al., 2012b, 2012a). Toxicity may be dose dependent or cumulative (Tarhini, 2013), and toxicities may be increased when these agents are given in combination with other systemic treatments or radiation therapy (Hodi et al., 2010; Lynch et al., 2012; Reck et al., 2013).

There has been variability in grades 3 and 4 toxicities between different classes of checkpoint inhibitors, with anti–PD-1 and anti–PD-L1 inhibitors demonstrating a better toxicity profile than ipilimumab (Brahmer et al., 2012; Davies, 2014b; Ibrahim et al., 2011; Sznol et al., 2013; Topalian et al., 2012b). This may be because CTLA-4 modulates early phases of T-cell activation in the immune response (Postow et al., 2015). In contrast, the PD-1/PD-L1 pathways limit T-cell activity at the time of an immune-inflammatory response, hence protecting normal tissues from autoimmunity (Topalian et al., 2012a). Anti–PD-1/PD-L1 agents act where T cells are directly interacting with tumor cells, therefore limiting exposure to normal tissue.

Treatment of irAEs differs from the management of AEs associated with cytotoxic agents. The irAEs are managed through adherence of specific guidelines (Table 4; Davies, 2014b; Kreamer, 2014; Rubin, 2012; Weber et al., 2012; Weber et al., 2015). Grading of toxicities using the Common Terminology Criteria for Adverse Events (CTCAE) manual facilitates better implementation of treatment recommendations for irAEs.

**Table 4:**
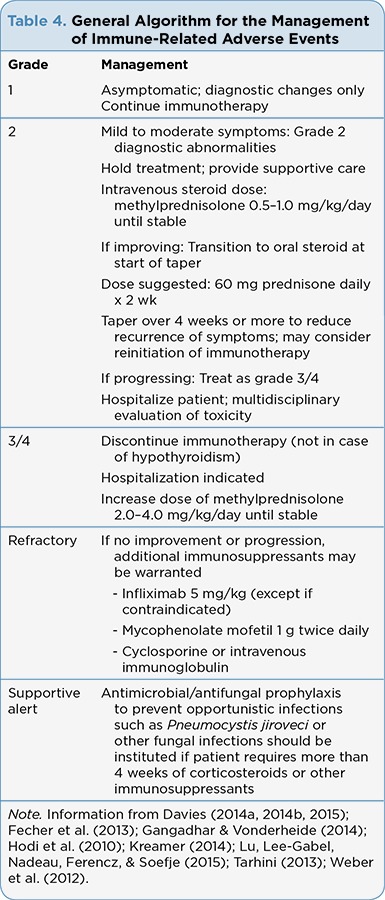
General Algorithm for the Management of Immune-Related Adverse Events

Generally, irAEs are managed by withholding immunotherapy. If irAEs are more severe, suppression of the immune system with corticosteroids may be necessary (Postow et al., 2015). It is important to remember that the AEs a patient experiences may not be related to the checkpoint inhibitor therapy and to rule out other potential causes. Dosing of checkpoint inhibitors may be delayed until resolution of irAEs. There are no dose reductions with checkpoint inhibitor therapy.

## ORGAN-SPECIFIC MANAGEMENT OF IMMUNE-RELATED ADVERSE EVENTS

Dermatologic toxicity is the most common irAE; it is seen in 47% to 68% of patients receiving ipilimumab (Wolchok et al., 2010; O’Day et al., 2010) and is less frequent in patients receiving anti–PD-1 agents. Toxicity includes pruritus, rash, erythroderma, vitiligo (hypopigmentation), macular rash, urticaria, mucositis, eosinophilic infiltrates, or lichenoid/spongiotic dermatitis. Severe toxicity such as Stevens-Johnson syndrome or toxic epidermal necrolysis is rare; however, there have been deaths reported with severe toxicity following ipilimumab (Lacouture et al., 2014; O’Day et al., 2010; Wolchok et al., 2010). Rash of the trunk and extremities is the most common presentation and typically occurs in the first month of treatment. It may be pink to red and may or may not be pruritic. It is important to point out that rash is seen less commonly with anti–PD-1 agents than with other checkpoint inhibitors (i.e., the CTLA-4 inhibitors).

Grade 1 rash is treated with supportive care including application of cool compresses, emollient lotions, or oatmeal baths. Diphenhydramine or hydroxyzine may help to reduce pruritus. Topical corticosteroids with low potency, such as triamcinolone cream or hydrocortisone cream, are used for grade 2 rash. Topical antibiotics may be required for open areas. Corticosteroids are recommended for grade 3 to 4 rash. Any new skin lesions should be evaluated by a dermatologist, because a biopsy may be necessary. Patients should be instructed to avoid sun exposure, which may exacerbate rash.

Gastrointestinal irAEs include bloating, cramps, gas, nausea, diarrhea, abdominal pain, and colitis. The onset of these irAEs is usually after two to three doses of a checkpoint inhibitor (i.e., 6–7 weeks after therapy has been initiated). Diarrhea may progress to colitis, which can lead to obstruction and perforation if left untreated (Fecher et al., 2013). Colitis is more commonly seen with anti–CTLA-4 agents and may be seen without diarrhea. Stool cultures, stool leukocytes, and *Clostridium difficile* titer should be obtained to rule out an infectious etiology of these AEs.

Grade 1 immune-mediated diarrhea and cramping can be managed with proton-pump inhibitors, antispasmodics, and antidiarrheal agents (e.g., dicyclomine [Bentyl], loperamide, and diphenoxylate/atropine), hydration, and dietary modifications. Dietary modifications include avoiding artificial sweeteners (e.g., sorbitol), dairy, caffeine, and foods that are spicy and/or high in fiber. Clinicians should also minimize medications that may contribute to diarrhea, such as antacids with magnesium.

If diarrhea continues for longer than 1 week or is at grade 2, oral steroids should be initiated. Budesonide (9 mg/day) may be effective in decreasing local inflammation. Data suggest that budesonide is not beneficial in the prophylactic setting (Weber et al., 2009), but no study has been done on its effect in patients who develop diarrhea while taking checkpoint inhibitors. Although its use may not be recommended, some clinicians do use budesonide for this purpose in practice.

Grade 3 to 4 diarrhea is managed with IV corticosteroids. A gastroenterology consult should be obtained. Abdominal ultrasound, computed tomography (CT), or colonoscopy (with biopsy) may be necessary to rule out peritonitis, colitis, or perforation. For refractory symptoms after 48 to 72 hours on high-dose steroids, infliximab at 5 mg/kg every 2 weeks, has been used unless contraindicated (Lemech & Arkenau, 2012; Minor, Chin, & Kashani-Sabet, 2009; Tarhini et al., 2010).

Pneumonitis is a potentially serious irAE and is more frequently seen with anti–PD-1 agents, specifically in patients with NSCLC, possibly due to chronic lung inflammation (Hamid et al., 2013b; Garon et al., 2013). Deaths due to pneumonitis occurred in early phase I studies of nivolumab (Topalian et al., 2012b). Presenting symptoms of pneumonitis are shortness of breath, cough, pleuritic chest pain, and/or fever. Baseline oxygen saturation, at rest and with ambulation, should be obtained for all patients prior to the start of checkpoint inhibitor treatment and with each subsequent visit. Subtle changes in oxygen saturation (i.e., hypoxia) are usually the first sign of pneumonitis.

If clinicians suspect pneumonitis, chest CT could help rule out other causes of this AE, such as infection, disease progression, pulmonary embolism, or effusion; however, suspicion should remain high in symptomatic patients, because radiographic findings can be variable. Bronchoscopy with biopsy may be needed to differentiate between infection and immune-mediated inflammation. Oxygen support, albuterol nebulizer, and corticosteroids are indicated for grade 2 or higher pneumonitis.

Hepatitis is usually asymptomatic and identified on liver function tests (LFTs). If symptoms are present, they are usually mild and consist of nausea or vague abdominal discomfort. The onset of this irAE is usually 9 to 10 weeks after therapy is initiated (Kim et al., 2013). Laboratory analysis of LFTs (i.e., aspartate aminotransferase, alanine aminotransferase, alkaline phosphatase, total and direct bilirubin) should be monitored at the start of therapy and with each visit.

Treatment should be held for grade 2 or greater LFT abnormalities, and the use of hepatotoxic agents (e.g., acetaminophen) should be avoided. Elevations in LFTs should be monitored. For hepatotoxicity of grade 3 or higher, corticosteroids should be initiated. If transaminase levels are not reduced within 48 hours, oral mycophenolate mofetil at 500 mg every 12 hours may be needed. Referral to a hepatologist is recommended for grade 3 to 4 toxicity, and a liver ultrasound, including the gallbladder, is suggested.

Autoimmune endocrinopathies have been reported with anti–CTLA-4 and anti–PD-1 checkpoint inhibitors. Toxicities include hypophysitis, hypothyroidism, hyperthyroidism, hyperpituitarism, and adrenal insufficiency. The occurrence of these irAEs is < 10%, with onset usually at 9 weeks after the initiation of checkpoint inhibitor therapy (Brahmer et al., 2012; Corsello et al., 2013; Hamid et al., 2013a, 2013b; Hodi et al., 2010; Ibrahim et al., 2011; Topalian et al., 2012b). Presenting symptoms of autoimmune endocrinopathies are nonspecific but may include fatigue, headache, vertigo, visual changes, changes in mental status, and hypotension. Most cases, however, are subclinical (Di Giacomo et al., 2010).

Thyroid-stimulating hormone should be assessed prior to treatment and on a monthly basis thereafter. Levels of hormones that regulate endocrine organs should be assessed as indicated. Consultation with an endocrinologist is recommended to evaluate patients suspected of having any of these toxicities. If hypophysitis is suspected, a pituitary scan or magnetic resonance imaging (MRI) of the brain should be obtained to rule out brain metastases (Corsello et al., 2013).

Treatment of endocrinopathies requires appropriate hormone replacement. Hypothyroidism is the most common endocrine irAE and is treated with thyroid hormone replacement. Immunotherapy treatment can continue while treating hypothyroidism. If symptomatic, hyperthyroidism is treated with beta-blockers; thyroid-suppression therapy may be required. In most cases, however, hyperthyroidism/thyroiditis converts to a hypothyroid state within a few weeks.

Hypophysitis (inflammation of the pituitary) occurred in 4.9% to 17% of patients across studies (Corsello et al., 2013). The median time to onset was 11 weeks after initiation of checkpoint inhibitor therapy and was more common in males. Magnetic resonance imaging of the brain may demonstrate enlargement of the pituitary gland, up to 60% to 100% enlarged. Hypophysitis may lead to hypopituitarism and adrenal insufficiency, which may be life-threatening. The damage may be irreversible, and patients may require lifetime replacement therapy with physiologic hydrocortisone doses (Dillard, Yedinak, Alumkal, & Fleseriu, 2010).

Inflammation (autoimmunity) occurs less frequently in the pancreas, kidneys, heart, neuromuscular system, and eyes. Pancreatitis may present with elevation of amylase or lipase or new-onset hyperglycemia. Blood glucose, amylase, and lipase levels should be monitored monthly in patients taking checkpoint inhibitors. Nephritis presents with gradual elevation of blood urea nitrogen and creatinine, with decreased creatinine clearance. Computed tomography of the abdomen and pelvis should be performed to rule out pancreatic obstruction and pre/post renal obstruction from lymphadenopathy. Consultations with a gastroenterologist and/or a nephrologist should be initiated, as biopsies may be needed as part of the evaluation.

Arrhythmia may develop as a result of pericarditis. A baseline electrocardiogram is suggested before therapy is initiated. A full cardiac evaluation for a patient suspected of having cardiac-related irAEs includes an echocardiogram and a cardiology consult.

Neuromuscular manifestations include muscle weakness, peripheral neuropathy, meningitis, myasthenia gravis, and Guillain-Barré syndrome (Bot, Blank, Boogerd, & Brandsma, 2013). Magnetic resonance imaging of the brain should be performed to rule out stroke or brain metastasis.

Ocular inflammation is infrequent with the use of checkpoint inhibitor therapy. Patients may present with itchy, watery eyes; photophobia; pain; dryness; and visual changes. Manifestations include conjunctivitis, uveitis, iritis, and scleritis. Patients in whom colitis develops may have a higher likelihood of uveitis. An ophthalmology consult should be obtained for patients with suspected ophthalmologic irAEs to rule out infection. Typically, these ocular irAEs are treated with corticosteroid eyedrops.

Health-care providers should have a low threshold for acting on symptoms, as time to recovery is facilitated by early identification and management (Fecher et al., 2013). The median time to recovery is 6 weeks. Corticosteroid administration should continue until toxicity is improved to grade 1 or resolved. Steroids should be tapered over at least 1 month to prevent relapse.

Once the irAE has resolved, retreatment can be considered; however, the decision to restart treatment should be made on an individual basis. If immunotherapy is restarted, clinicians should closely monitor patients for recurrence of toxicity. The use of corticosteroids does not appear to negatively impact outcomes of treatment with checkpoint inhibitors (Amin et al., 2009; Harmankaya et al., 2011).

The following two case studies are provided to help illustrate how some of this information on managing toxicities can be put into practice.

## CASE STUDY 1

Mrs. S is a 54-year-old woman with metastatic NSCLC. Computed tomography scan at diagnosis revealed a mass measuring 6.2 ✕ 3.4 cm in the right upper lobe and right adrenal metastasis. An endobronchial ultrasound biopsy of the right upper lobe confirmed adenocarcinoma. The tumor did not express *EGFR* mutation or *ALK* rearrangement. Mrs. S was initially treated with a platinum-based chemotherapy regimen. Following four cycles of therapy, disease progression developed with multiple small hepatic metastases.

At her next appointment, Mrs. S was asymptomatic, physically active, and in no acute distress. Vital signs were heart rate 64 beats/minute, blood pressure was 122/78 mm Hg, and respiratory rate was 14 breaths/minute. Oxygen saturation was 98% on room air and with ambulation. On physical examination, breath sounds were clear bilaterally. Cardiac examination demonstrated a regular heart rate and rhythm. Laboratory results, including LFTs, were all within normal limits.

The decision was made to initiate treatment with nivolumab at 3 mg/kg every 2 weeks. At cycle 8 of treatment, Mrs. S was scheduled to meet with the advanced practitioner (AP). She reported a mild, nonproductive cough and mild dyspnea; as a result, she reported a decline in activity over the past week. She denied fever and chest pain. Physical examination revealed decreased breath sounds at the right lung base and no adventitious sounds. Complete blood cell count and comprehensive metabolic panel results were within normal limits. Oxygen saturation was 98% at rest and decreased to 92% with ambulation.

The AP ordered a CT scan to evaluate potential causes (i.e., pulmonary embolism, infection, progression of disease, pneumonitis). The CT scan demonstrated ground-glass opacities in the right middle lobe and right lower lobe, suggestive of infection vs. pneumonitis. A bronchoscopy was ordered, and biopsy results confirmed pneumonitis. Treatment with nivolumab was withheld. She was monitored closely for symptoms and instructed to contact the clinic with any change.

Three weeks later, Mrs. S reported her cough had resolved and she had resumed all activities. Oxygen saturation improved to 98% with ambulation. A repeat CT scan indicated complete resolution of the ground-glass opacity, as well as a decrease in the size of the primary right upper lobe tumor. She was restarted on nivolumab therapy. At cycle 24, CT scans demonstrated a complete response to treatment in the right upper lobe, adrenal, and hepatic metastases. She had no recurrence of pneumonitis symptoms throughout the course of treatment.

## CASE STUDY 2

Mr. K is a 72-year-old man who presented with a changing "black mole" on the left upper thigh with associated firmness in the left groin. Biopsy results revealed melanoma. Staging CT scans confirmed metastases in the lungs and liver. Mr. K was otherwise asymptomatic. His medical history was positive for hyperlipidemia and hypertension, which were treated and controlled with a statin and hydrochlorothiazide, respectively. Vital signs were within normal limits.

Physical examination revealed clear breath sounds bilaterally, a regular cardiac rate and rhythm, active bowel sounds, a nontender abdomen, and no hepatomegaly. A 3-cm nodule was palpated in the left inguinal region. No other adenopathy was appreciated. Baseline laboratory results, including a complete blood cell count and comprehensive metabolic panel, were within normal limits.

The decision was made to initiate treatment with ipilimumab (3 mg/kg) and nivolumab (1 mg/kg). Staging scans after four cycles demonstrated a significant tumor response, with reduction in lung and liver lesions. The AP obtained pretreatment bloodwork, which indicated grade 3 elevation of transaminases and grade 2 elevation of creatinine. The AP completed a medication reconciliation, and Mr. K confirmed that he had not had changes to medications. Mild abdominal tenderness, normal active bowel sounds, and no hepatomegaly were found on physical examination. The left inguinal node was no longer palpable.

Further treatment with checkpoint inhibitors was withheld. The statin and hydrochlorothiazide also were withheld. Mr. K was started on methylprednisolone at 1.0 mg/kg for 5 days, and the AP monitored him weekly. He remained asymptomatic during that period. The creatinine level improved to normal, and transaminase levels improved to grade 2. The steroids were transitioned to oral prednisone and tapered over 4 weeks. Transaminase levels improved to within normal limits. Mr. K was restarted on nivolumab as single-agent therapy every 2 weeks. (The statin and hydrochlorothiazide were also restarted.) Staging scans continued to demonstrate a positive response to treatment.

## ROLE OF THE ADVANCED PRACTITIONER

Advanced practitioners play a critical role in caring for patients treated with checkpoint inhibitors. It is essential for APs to be aware of the mechanism of action of these agents, patterns of response seen with this type of therapy, and presentation of irAEs related to these agents to ensure timely and successful treatment (Andrews & Holden, 2012; Davies, 2014b; Kreamer, 2014; Rubin, 2012). Rapid evaluation/diagnostics and treatment are essential for optimal management and prevention of end-organ disease, and treatment of irAEs requires a multidisciplinary approach.

Advanced practitioners are involved in the identification of patients who might be candidates for immunotherapy. Patients who may be suitable for checkpoint inhibitor therapy include those with a good performance status and less aggressive or lower tumor burden. Patients with more aggressive tumor burden may need other types of therapies, with a more rapid onset of action.

Advanced practitioners must obtain a full medical history and physical examination and review baseline symptoms and laboratory values prior to the start of therapy. Vital signs should include oxygen saturation at rest and with ambulation to assess stress response. Medications, including over-the-counter drugs and herbal supplements, should be documented and evaluated to minimize the use of hepatotoxic and nephrotoxic agents. Patients should be screened for a history of autoimmune diseases. Patients with autoimmune diseases were excluded from participation in the clinical trials of checkpoint inhibitors because theoretically the treatment could exacerbate their conditions.

Clinicians will need to evaluate the risk of treating patients with these conditions:

- Neuromuscular: multiple sclerosis, myasthenia gravis
- Gastrointestinal: celiac disease, Crohn’s disease, ulcerative colitis
- Rheumatoid: arthritis, Sjögren syndrome, systemic lupus erythematosus
- Endocrine: Addison disease, Graves’ disease, Hashimoto thyroiditis, type 1 diabetes mellitus
- Hematologic: pernicious anemia
- Prior organ transplant (patients may be on heavy immunosuppressants)
- History of organ damage from chronic infection, disease, or drugs

Patients must then be monitored at each visit for potentially serious AEs. Adverse events should be graded according to the CTCAE criteria. This provides a foundation for the implementation of treatment algorithms and supports communication among all members of the health-care team. Screening for irAEs must also be done during all triage-related telephone calls. Developing telephone triage guidelines can be instrumental to ensure prompt identification and management of adverse events. Use of treatment algorithms, educational tools, and checklists also can be of benefit.

Checkpoint inhibitors have varying administration schedules. Premedication, especially with steroids, is not recommended. In the event of irAEs, treatment may be delayed, but there are no dose reductions.

Patient education is critically important in the management of patients considered for immunotherapy (Table 5). Patients and their families/caregivers are essential members of the health-care team. They should be instructed on the mechanism of action, expected time to response, response patterns, and potential irAEs of checkpoint inhibitors prior to the start of therapy, and this information should be reinforced at each visit. Patients must be instructed to report any symptoms to the oncology team as soon as they occur. They should be reassured that irAEs are infrequent, treatable, and respond well to steroid therapy.

**Table 5:**
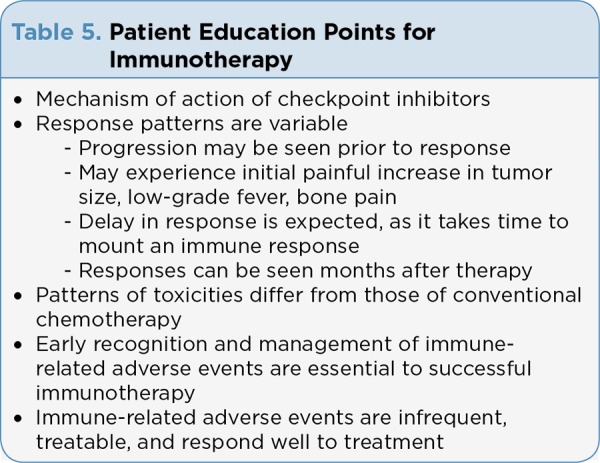
Patient Education Points for Immunotherapy

If receiving steroids, patients must follow a taper schedule closely over at least 1 month. Early discontinuation of steroids can predispose patients to a relapse or flare of symptoms. Immunosuppression for irAEs usually does not have a negative impact on outcomes.

Advanced practitioners should reassure patients that if identified early, irAEs can usually be successfully managed. Once the irAEs have been managed, patients may be restarted on treatment, even if therapy needed to be stopped/delayed for a time.

Patients should be instructed to use effective contraception throughout treatment and for at least 6 months following therapy, because antibodies are known to cross the placental barrier. If a patient is receiving hormone-replacement therapy, a medical alert bracelet should be worn. This will alert health-care providers in the event that stress doses of replacement are needed in emergent health crises. Patients should discuss the addition of any new medications, including over-the-counter drugs and herbal supplements, with the treating team to ensure there are no contraindications.

## CONCLUSION

Checkpoint inhibitors have changed the clinical landscape for patients diagnosed with MM, NSCLC, and RCC. Patients with these diseases have historically had poor survival outcomes. Durable, long-term responses have been demonstrated in patients treated with checkpoint inhibitors. There is some indication that combination treatment with two checkpoint inhibitors or a checkpoint inhibitor combined with chemotherapy or radiation therapy may improve outcomes further.

It is essential for all practitioners to be familiar with the patterns of toxicities of these therapies. Early identification and treatment can minimize risk for advanced toxicities and long-term complications. In many cases, prompt treatment provides an opportunity for patients to continue on treatment. Advanced practitioners play a critical role in maintaining therapy for these patients.

## Method of Participation and Request for Credit

There are no fees for participating and receiving CNE credit for this activity. During the period July 12, 2016, through July 11, 2017, participants must read the learning objectives and faculty disclosures and study the educational activity. 

If you wish to receive credit/acknowledgment for completing this activity, please visit http://axismeded.com/content/JADPRO and complete the post-test and evaluation. Upon registering and successfully completing the post-test with a score of 75% or better and the activity evaluation, your certificate will be available immediately. 
